# Identification and validation of a genomic mutation signature as a predictor for immunotherapy in NSCLC

**DOI:** 10.1042/BSR20220892

**Published:** 2022-11-25

**Authors:** Zemin Wang, You Ge, Han Li, Gaoqiang Fei, Shuai Wang, Pingmin Wei

**Affiliations:** Department of Epidemiology and Health Statistics, School of Public Health, Southeast University, Nanjing, Jiangsu, China

**Keywords:** Biomarker, Genomic mutation signature, Immune checkpoint inhibitor, Immunotherapy, Non-small cell lung cancer

## Abstract

Currently, the benefits of immune checkpoint inhibitor (ICI) therapy prediction via emerging biomarkers have been identified, and the association between genomic mutation signatures (GMS) and immunotherapy benefits has been widely recognized as well. However, the evidence about non-small cell lung cancer (NSCLC) remains limited. We analyzed 310 immunotherapy patients with NSCLC from the Memorial Sloan Kettering Cancer Center (MSKCC) cohort. Lasso Cox regression was used to construct a GMS, and the prognostic value of GMS could be able to verify in the Rizvi cohort (*N*=240) and Hellmann cohort (*N*=75). We further conducted immunotherapy-related characteristics analysis in The Cancer Genome Atlas (TCGA) cohort (*N*=1052). A total of seven genes (*ZFHX3, NTRK3, EPHA7, MGA, STK11, EPHA5, TP53*) were identified for GMS model construction. Compared with GMS-high patients, patients with GMS-low had longer overall survival (OS;* P*<0.001) in the MSKCC cohort and progression-free survival (PFS; *P*<0.001) in the validation cohort. Multivariate Cox analysis revealed that GMS was an independent predictive factor for NSCLC patients in both the MSKCC and validation cohort. Meanwhile, we found that GMS-low patients reflected enhanced antitumor immunity in TCGA cohort. The results indicated that GMS had not only potential predictive value for the benefit of immunotherapy but also may serve as a potential biomarker to guide clinical ICI treatment decisions for NSCLC.

## Introduction

Global cancer data for 2020 has shown that lung cancer remained the malignant tumor with the highest mortality rate (18%) worldwide as well as the incidence rate (11.4%) ranked only second to female breast cancer (11.7%) [1]. Approximately 80–85% of those cases are currently classified as non-small cell lung cancer (NSCLC), of which 5-year survival rates were less than 15–20% [2]. Two of the main dominant histological phenotypes of NSCLC include lung adenocarcinoma (LUAD; 50%) and lung squamous cell carcinoma (LUSC; 40%) [3]. Recently, immune checkpoint inhibitors (ICIs) have been applied to NSCLC treatment in order to transform the therapeutic landscape for the condition [4]. In fact, substantial advances in clinical treatment have not provided the equivalent benefit of ICIs among the majority of patients, and the results of the recent clinical trial emphasized the necessity of effective selection for biomarker-based patients [5]. Therefore, it is particularly important to identify and develop potential predictive biomarkers that can be used to predict the efficacy of ICI in dominant populations.

To date, increasing numbers of biomarkers have been confirmed to predict the benefits of immunotherapy. As predictive biomarkers for the ICIs, tumor mutation burden (TMB) and programmed death ligand 1 (PD-L1) expression have been prospectively verified in the randomized controlled trials (RCTs) of NSCLC [6,7]. Nevertheless, TMB and PD-L1 are not beneficial for all NSCLC patients, and it is still required to explore novel biomarkers to maximize clinical benefits [8,9]. At present, a growing body of studies has demonstrated that genomic mutation signatures (GMS) have great potential in predicting tumor prognosis. For example, Jiao et al. established a six-gene-based signature (including genes *RNF43, CREBBP, CDKN2A, TP53, SPEN*, and *NOTCH3*), which was not only a powerful predictive factor of immunotherapy efficacy for gastrointestinal cancer but also may be regarded as the potential biomarker to guide clinical treatment [10]. Similarly, Bai et al. developed another eight-gene-based prognostic model (*HGF, KRAS, EGFR, PTPRD, STK11, KMT2C, SMAD4*, and *TP53*) to predict the response of nonsquamous NSCLC to PD-1 inhibitors [11]. In addition, Pan et al. also constructed a mutation classifier (*TP53, PIK3CA*, and *ATM*) to predict the benefits of ICI treatment in bladder cancer patients [12]. Therefore, we can deeply explore the genomic data and identify novel GMSs, so as to guide prognosis stratification and personalized treatments of patients.

In the present study, we integrated the immunotherapy cohort of NSCLC to develop and validate a novel GMS to predict immunotherapy responsiveness. Additionally, we further conducted immunotherapy-related characteristics analysis in The Cancer Genome Atlas (TCGA) cohort as well. Collectively, our results suggested the seven-gene signature could be served as a powerful predictive indicator of immunotherapy and may guide clinical ICI treatment decisions as a potential biomarker in NSCLC.

## Materials and methods

### Study design and samples

**Figure 1:**
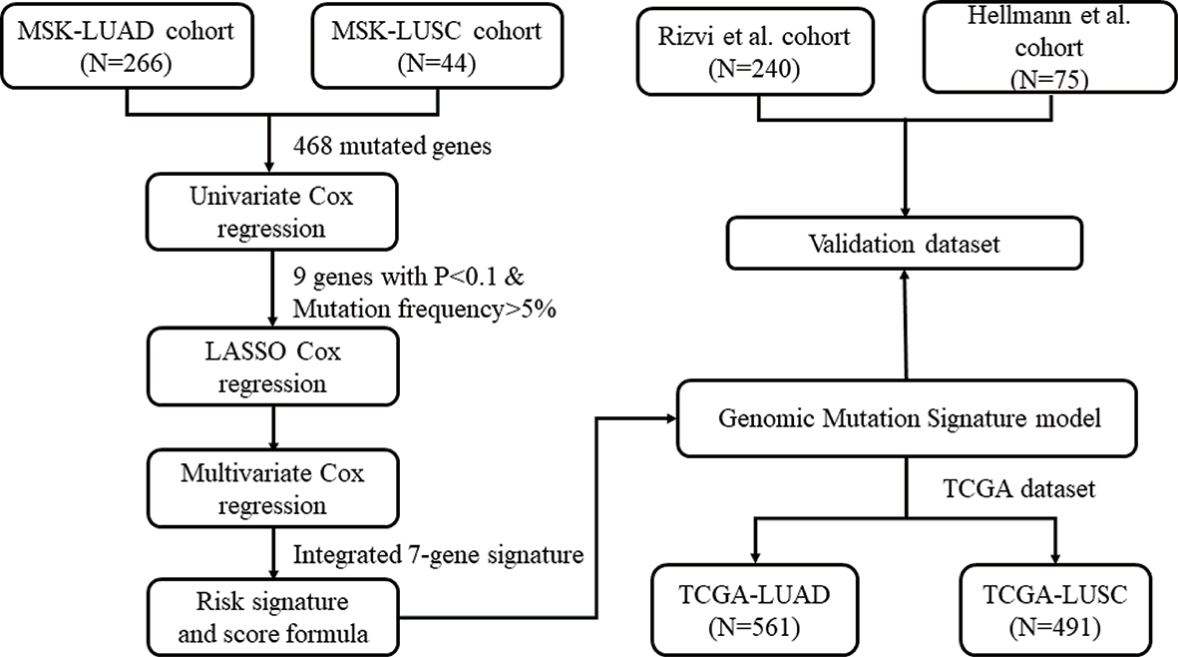
The flowchart of the study design

In the present study, a three-step approach was (discovery cohort, validation cohort, and TCGA dataset) applied for developing and validating a GMS for the predictive ability of immunotherapy among patients with NSCLC. The flow chart of the study design was illustrated in Figure 1. In order to evaluate the relationship between gene mutation and the efficacy of ICI, we obtained the clinical and genomic data of advanced cancer patients who have been treated with ICI in the Memorial Sloan Kettering Cancer Center (MSKCC) cohort (http://www.cbioportal.org/) [13]. A total of 310 NSCLC patients were identified as the discovery cohort, including 266 with LUAD and 44 with LUSC. Then, we adopted the clinical cohorts, which were treated by ICIs in two published cohorts during the subsequent validation phase. The cohort of 75 patients with advanced NSCLC who received combined immunotherapy was collected from the Hellmann et al.’s study [14]. The cohort of 240 patients with advanced NSCLC treated with anti-PD-1 or anti-PD-L1 therapy was obtained from Rizvi et al.’s study [15]. The mutation and clinical data of each sample in the discovery and validation cohorts were collected from cBioPortal and previous studies [13–15]. In addition, the TCGA cohort was used to explore whether GMS can be considered as a useful indicator for tumor immune microenvironment characteristics. The data of TCGA-LUAD and TCGA-LUSC were obtained from TCGA (https://portal.gdc.can/cer.gov/). The corresponding clinical data were acquired from UCSC Xena (http://xena.ucsc.edu/). Patients who were involved in the three cohorts with incomplete clinical information and mutation data would be excluded.

### Clinical outcomes

The clinical outcomes of the present study mainly included progression-free survival (PFS), objective response rate (ORR), overall survival (OS), and durable clinical benefit (DCB). PFS was assessed from the date of initiation of immunotherapy to the time of progress or death due to any causes. ORR was determined based on Response Evaluation Criteria in Solid Tumors (RECIST) version 1.1 [16]. OS refers to the time from random grouping to death, which was caused by any reasons. Complete response (CR), partial response (PR), or stable disease (SD) lasting more than 24 weeks was recognized as a durable clinical benefit (DCB); SD or progressive disease (PD) lasting less than 24 weeks was assumed as no durable benefits (NDB) [17]. Patients who did not develop symptoms and were censored before 24 weeks of follow-up should be defined as not evaluated (NE).

### Construction of the GMS

First, univariate Cox regression analyses were performed on the relationship between prognosis-related gene mutations (mutation frequency > 5%) and the survival of 310 patients in two cohorts. Then, we conducted a least absolute shrinkage and selection operator (LASSO) Cox regression analysis on genes with a *P-*value less than 0.1 determined by univariate Cox regression [10]. The multivariate Cox regression analysis was used to create the optimal signature. We used the Survminer R package to generate the optimal cutoff value to divide the patients into the GMS-low group and GMS-high group. The calculation formula of risk score showed as follows:

GMS score = (β_1_×mutation status of Gene_1_) + (β_2_×mutation status of Gene_2_) +…+ (β_n_ × mutation status of Gene_n_). Gene mutation status (1 or 0) was coded by mutant and wild-type genes. β was the regression coefficient generated in the multivariate Cox regression analysis.

### Immunotherapy-related characteristics analysis

As a representative of the expression in tumor-specific neoantigen, TMB may trigger the immune response. TMB was determined as the total number of nonsynonymous somatic mutations per megabase (Mb) in the genome [18]. For WES data in TCGA dataset, 38 Mb was used as the assessed exon size [19]. For samples in both of MSKCC cohort and validation cohort, TMB data were derived from the Memorial Sloan Kettering Integrated Mutation Profiling of Actionable Cancer Targets (MSK-IMPACT).

We characterized tumor immune activation according to studies of immune-related analysis that were previously published, including cytolytic activity (CYT) score [20], inflammation signature score [21], immunologic constant of rejection (ICR) score [22], IFN-γ signaling score [23], antigen processing machinery (APM) score [24], CD8^+^T effector score [25], and the activity of 13 immune-related pathways [26]. These indicators were confirmed to correlate with the efficacy of immunotherapy. The CYT score was determined based on granzyme A (GZMA) and perforin 1 (PRF1) expression [27]. The method of single-sample gene set enrichment analysis (ssGSEA) in the GSVA R package was used to quantify the above indicators [28]. The IFN-γ signaling score was performed via the gene sets of KEYNOTE-012 [29] and POPLAR [30] in ICIs-treated clinical trials. In addition, we also applied the ssGSEA for calculating the infiltration scores of 16 immune cells to estimate the abundance of tumor-infiltrating lymphocytes [28].

### Statistical analysis

Statistical analyses were performed using R v. 4.1.1, GraphPad Prism (V.8.0.2), and SPSS V.26.0 (SPSS). Cox regression analysis was performed to establish the GMS. The optimal cut-off value of GMS was conducted by the Survminer R package. The ORR and DCB in different subgroups were analyzed by the χ² test or Fisher’s exact test. Kaplan–Meier method and log-rank test were applied to calculate PFS and OS. The Man–Whitney U test or Kruskal–Wallis test was used to compare differences between two independent subgroups. All reported *P*-values less than 0.05 were considered statistically significant.

## Results

### Construction of the GMS

**Figure 2:**
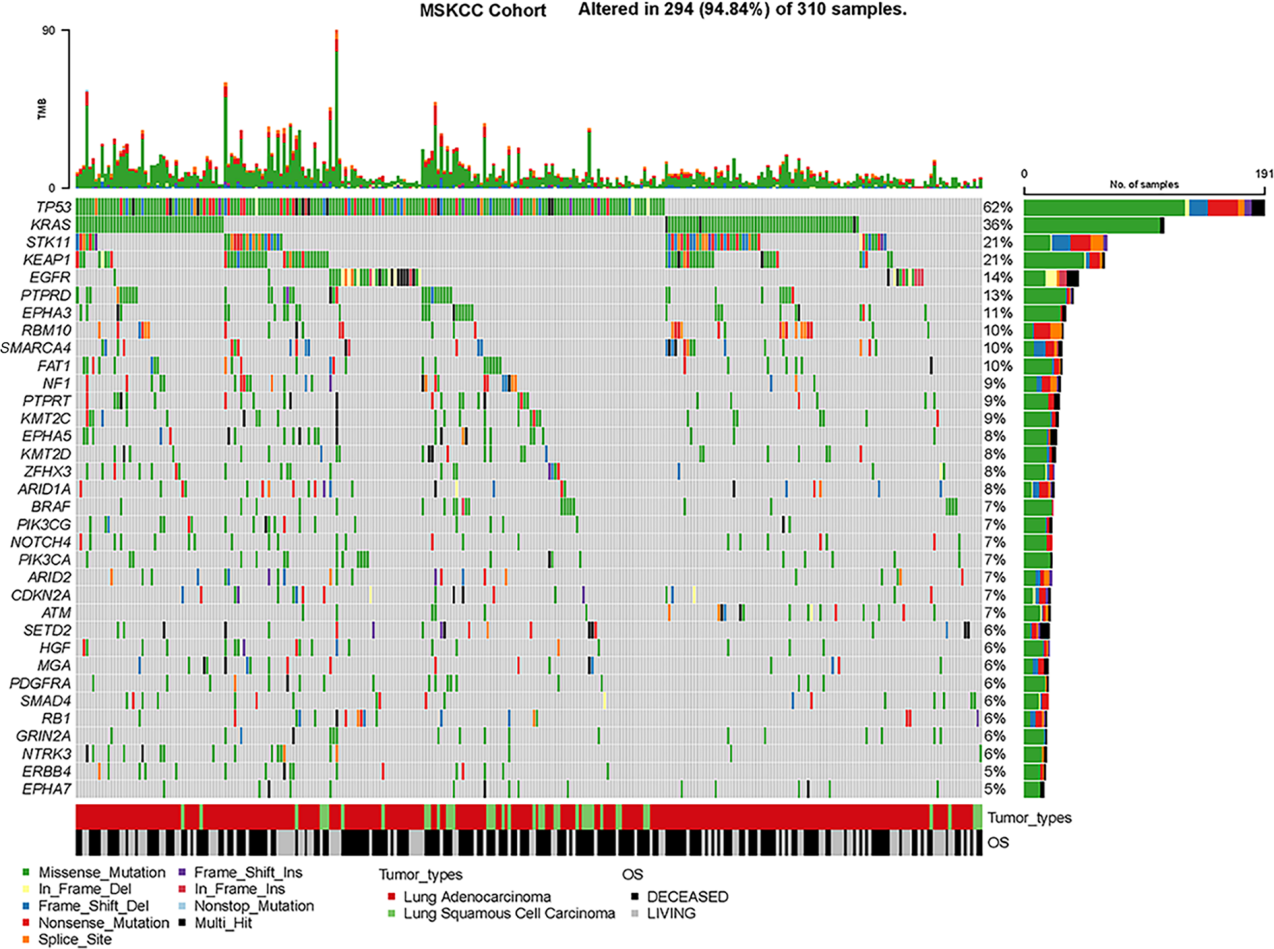
Oncoplot of the mutated genes Assessment of the frequency and pattern of mutations in patients with NSCLC from the MSKCC cohort. The mutation genes of 310 patients with NSCLC in this cohort were analyzed. Genes were listed by mutation frequency > 5%.

**Figure 3:**
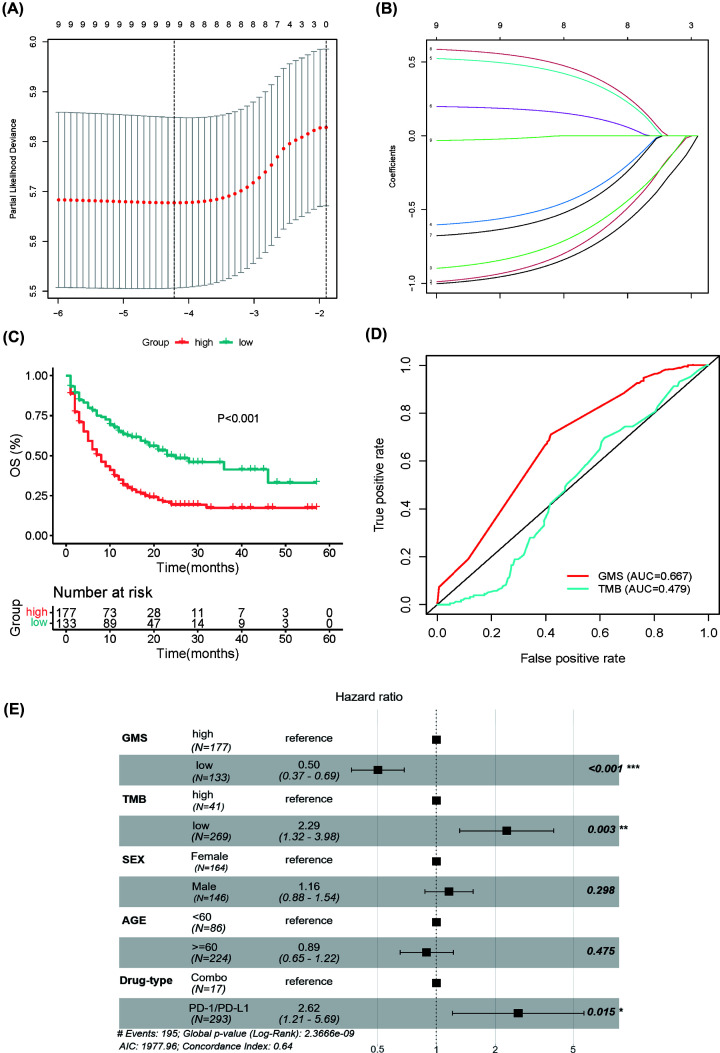
Construction of the GMS (**A**) The LASSO regression was performed for variable selection. (**B**) LASSO coefficient profiles of the nine candidate genes. (**C**) Kaplan–Meier curves for the OS of patients in the GMS-high group and GMS-low group in the MSKCC cohort. (**D**) The ROC curve measuring the predictive value of GMS and TMB in the MSKCC cohort. (**E**) Multivariate Cox analysis for OS in the MSKCC cohort.

Our study developed a predictive model named GMS based on MSKCC cohort, which included 310 lung cancer patients receiving ICI treatment (MSKCC-LUAD cohort, *N*=266; MSKCC-LUSC, *N*=44). The top 5% mutation frequency and pattern of mutations among patients with LUAD and LUSC from the MSKCC cohort were presented in Figure 2. First, a univariate Cox regression model was performed to select prognosis-related gene mutation (cases with mutation frequency > 5%). The genes with a *P*-value less than 0.1 in univariate Cox analyses were introduced into LASSO Cox regression (Figure 3A,B). After that, these candidate mutation genes were calculated by a multivariate Cox regression model to predict the OS of the MSKCC training cohort. Finally, totally of seven genes (*ZFHX3, NTRK3, EPHA7, MGA, STK11, EPHA5, TP53*) were identified to form the optimal model. Based on the mutation status of the seven genes (1 or 0) weighted by their regression coefficient, the GMS risk model was calculated for each patient (Table 1). GMS score = (0.652 × TP53) – (1.052 × ZFHX3) – (1.111 × NTRK3) – (0.974 × EPHA7) – (0.629 × MGA) + (0.654 × STK11) – (0.664 × EPHA5). In the calculation formula, the mutant genes were coded as 1 and the wild-type genes were coded as 0. The patients were divided into the GMS-high group and GMS-low group by optimal cutoff value 1, which was calculated by the Survminer R package. Compared with the GMS-high group, patients in the GMS-low group (*P*<0.001) had longer OS (Figure 3C). In order to appraise the sensitivity and specificity of predictions that identified GMS, receiver operating characteristic (ROC) curves were plotted as well as area under the curve (AUC) values were calculated. The ROC curve results illustrated that GMS performed better predictor ability (AUC = 0.667) compared with TMB (AUC = 0.479) in the MSKCC cohort (Figure 3D). In addition, GMS was considered as an independent prognostic factor in the MSKCC cohort. Table 2 provided the clinical characteristics between GMS-low and GMS-high patients of the MSKCC cohort. After multivariate adjustment of clinicopathological factors, GMS, TMB, and drug-type remained the powerful and independent prognostic factors (GMS: HR 0.50, 0.37–0.69, *P*<0.001; TMB: HR 2.29, 1.32–3.98, *P*=0.003; Drug-type: HR 2.62, 1.21–5.69, *P*=0.015) for OS (Figure 3E).

**Table 1:** Multivariable Cox regression analysis of candidate mutation genes in the MSKCC cohort

Variable	B	HR	95% CI	*P-*value
ZFHX3	−1.052	0.349	0.152 to 0.802	0.013
NTRK3	−1.111	0.329	0.132 to 0.822	0.017
EPHA7	−0.974	0.377	0.138 to 1.030	0.057
MGA	−0.629	0.533	0.256 to 1.110	0.092
STK11	0.654	1.924	1.345 to 2.752	<0.001
EPHA5	−0.664	0.515	0.276 to 0.962	0.037
TP53	0.652	1.919	1.391 to 2.647	<0.001

Abbreviations: B, regression coefficient; CI, confidence interval; HR, hazard ratio.

**Table 2:** Clinical characteristics between GMS-low and GMS-high patients of the MSKCC cohort

Characteristics	Classification	GMS-low	GMS-high	*χ*²	*P*-value
Age				0.291	0.590
	≥60	94	130		
	<60	39	47		
Sex				0.700	0.403
	Male	59	87		
	Female	74	90		
Drug type				3.490	0.062
	PD-1/PD-L1	122	171		
	Combo	11	6		
Cancer type				0.381	0.573
	LUAD	116	150		
	LUSC	17	27		

Abbreviations: LUAD, lung adenocarcinoma, LUSC, lung squamous cell carcinoma.

### Validation of the predictive value of GMS

**Figure 4:**
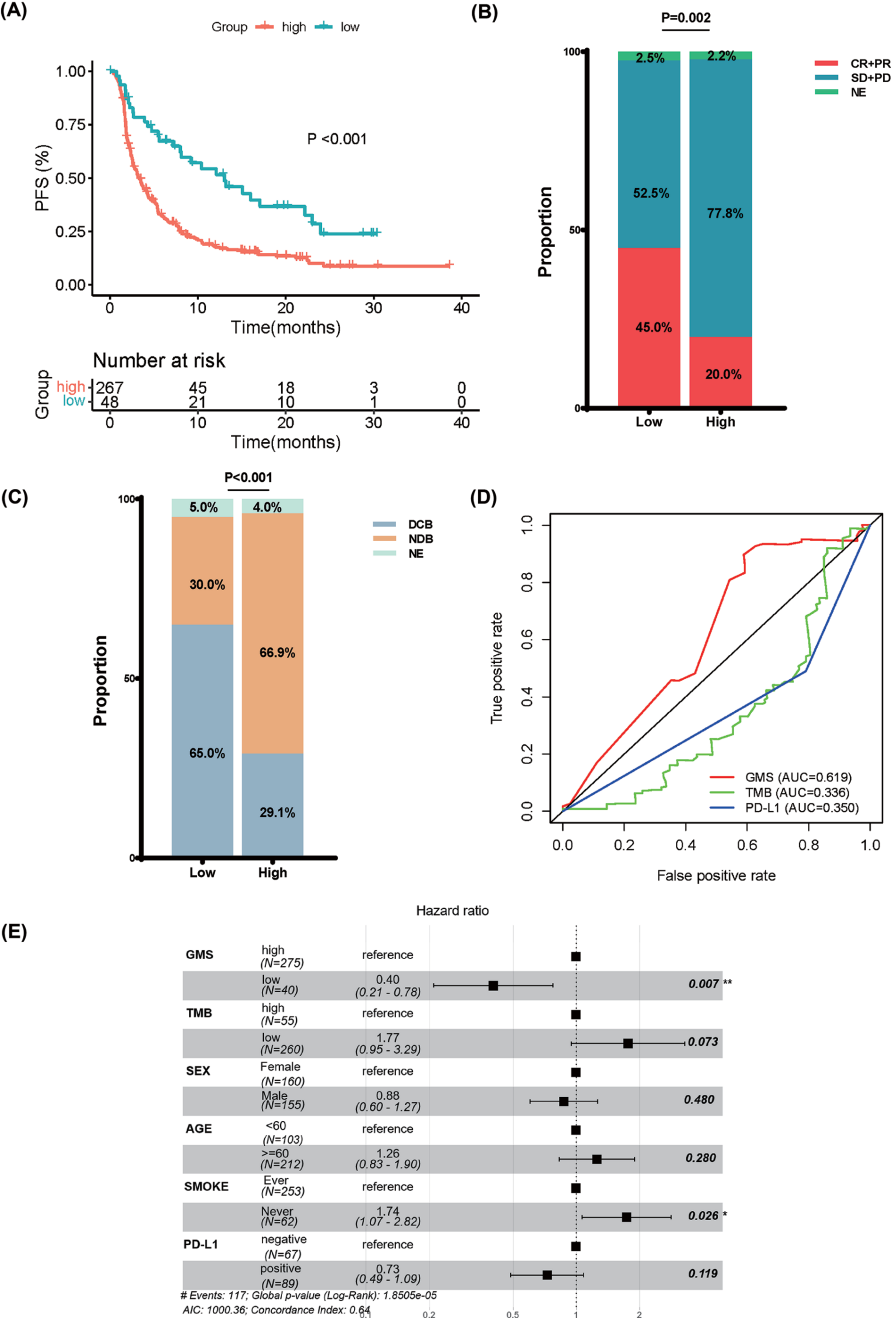
Validation of the predictive value of GMS (**A**) Kaplan–Meier curves for the PFS of patients in the GMS-high group and GMS-low group in the Rizvi and Hellman cohorts. (**B**) The proportion of ORR for patients with GMS-low and GMS-high groups in the Rizvi and Hellman cohorts. (**C**) The proportion of DCB for patients with GMS-low and GMS-high groups in the Rizvi and Hellman cohorts. (**D**) ROC curves measuring the predictive value of GMS, TMB, and PD-L1 in the Rizvi and Hellman cohorts. (**E**) Multivariate Cox analysis for PFS in the Rizvi and Hellman cohorts.

In order to further validate the prognostic ability of the GMS classifier, we integrated two previously published independent cohorts of NSCLC patients received ICIs treatment (Rizvi et al. cohort with 240 patients and Hellman et al. cohort with 75 patients). The patients in the validation cohort were divided into the GMS-high group and GMS-low group base on the optimal cutoff value. Compared with patients in GMS-high group, PFS was detected among patients of GMS-low group (*P*<0.001) (Figure 4A). The proportion of objective response (CR/PR) in GMS-low patients was over double than that in GMS-high patients (45% vs. 20%, *P*=0.002) (Figure 4B). The rate of DCB in GMS-low patients was significantly higher than that in GMS-high patients (65.0% vs. 29.1%, *P*<0.001) (Figure 4C). Furthermore, ROC analysis of the validation cohort indicated that GMS (AUC = 0.619) exhibited better predictive value compared with TMB (AUC = 0.336) and PD-L1 (AUC = 0.350) (Figure 4D). The clinical distribution characteristics between GMS-low and GMS-high patients in the Rizvi and Hellman cohorts were listed in Table 3. After multivariate Cox regression excluded other confounding factors, GMS and Smoke (GMS: HR 0.40, 0.21–0.78; *P*=0.007; Smoke: HR 1.74, 1.07–2.82, *P*=0.026) were independent predictors of PFS (Figure 4E). Overall, GMS can be used as a powerful predictor of the outcome of immunotherapy for NSCLC.

**Table 3:** Clinical distribution characteristics between GMS-low and GMS-high patients in the Rizvi and Hellman cohorts

Characteristics	Classification	GMS-low	GMS-high	*χ*²	*P*-value
Age				0.110	0.740
	≥60	26	186		
	<60	14	89		
Sex				0.324	0.569
	Male	18	137		
	Female	22	138		
Smoke				0.635	0.425
	Ever	34	219		
	Never	6	56		
PD-(L)1				3.268	0.195
	Positive	16	73		
	Negative	8	59		
	NE	16	143		
Best overall response				12.434	**0.002**
	CR/PR	18	55		
	SD/PD	21	214		
	NE	1	6		
Durable clinical benefit				21.111	**<0.001**
	DCB	26	80		
	NDB	12	184		
	NE	2	11		

Abbreviations: *CR*, complete response, *DCB*, durable clinical benefit, *NDB*, no durable benefit, *NE*, not evaluable, *PD*, progressive disease, *PD-(L)1*, programmed cell death-1 or programmed death-ligand 1, *PR*, partial response, *SD*, stable disease.

### Comparison of the immune activation characteristics of GMS in TCGA cohort

**Figure 5:**
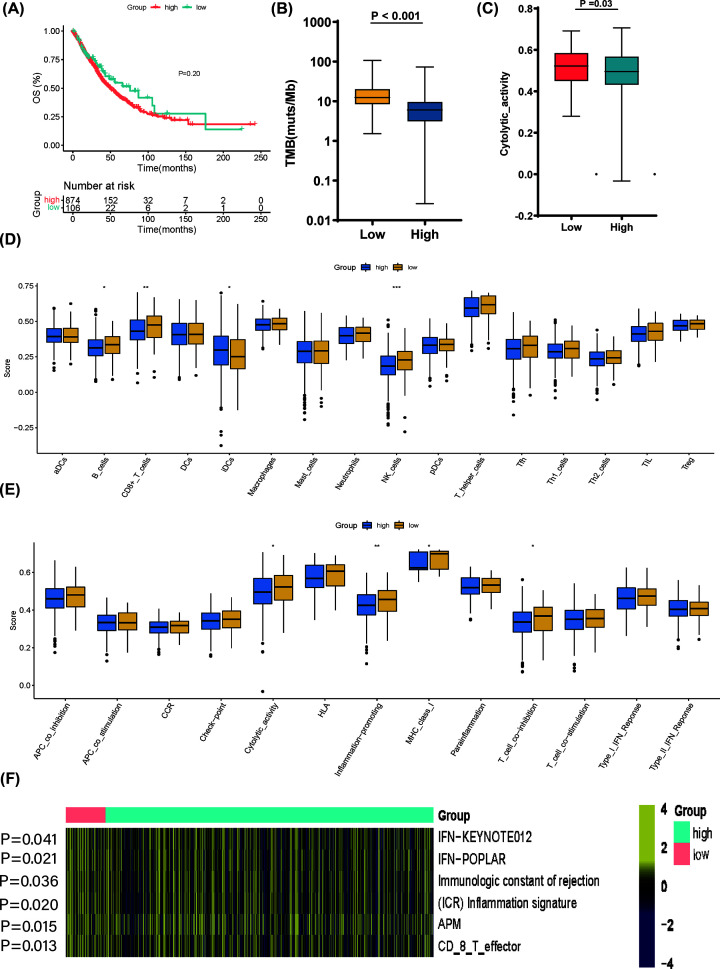
Comparison of the activation characteristics of GMS in TCGA cohort (**A**) Kaplan–Meier curves for the OS of patients in the GMS-high group and GMS-low group in the TCGA cohort. (**B**) Comparison of nonsynonymous mutations between the GMS-low and GMS-high groups in the TCGA cohort. (**C**) Comparison of cytolytic score between the GMS-low and GMS-high groups in the TCGA cohort. (**D**) Comparison of the enrichment scores of 16 types of immune cells between the GMS-low group (yellow box) and GMS-high group (blue box) in the TCGA cohort. (**E**) Comparison of the enrichment scores of 13 immune-related pathways between the GMS-low group (yellow box) and GMS-high group (blue box) in the TCGA cohort. *P*-values were showed as: ***P<0.001, **P<0.01, *P<0.05. (**F**) Heatmap of immune-related signatures between the GMS-low and GMS-high groups in the TCGA cohort.

According to the above observations, we assumed that GMS would be an indicator of tumor immune microenvironment characteristics for NSCLC patients. For subsequent analysis, we combined TCGA-LUAD (*N*=561) and TCGA-LUSC (*N*=491) cohorts. The TCGA cohort was divided into GMS-high and GMS-low based on the GMS risk model. As for OS analysis, Kaplan–Meier survival curves showed no significant differences were identified between GMS-low patients and GMS-high patients (*P*=0.20) in TCGA cohort (Figure 5A). Tumors with GMS-low showed remarkably more nonsynonymous mutations than those with GMS-high (*P*<0.001) (Figure 5B). The CYT score calculated by the ssGSEA method of the expression of GZMA and PRF1 was also higher among GMS-low patients (*P*=0.03) (Figure 5C). Using the ssGSEA methodology, we further evaluated the enrichment scores of 16 immune cells and the activity of 13 immune-related pathways between the GMS-high and GMS-low groups in TCGA cohort. In TCGA cohort, the immune infiltration level of the GMS-high group was commonly lower than that of the GMS-low group, especially B cells (*P*=0.042), CD8 ^+^ T cells (*P*=0.010), and NK cells (*P*<0.001). Moreover, induced dendritic cells (iDCs) have higher expression in the GMS-high group (*P*=0.037) (Figure 5D). In addition, four immune pathways showed lower activity in the GMS-high group compared with the GMS-low group in TCGA cohort, including CYT (*P*=0.030), Inflammation-promoting (*P*=0.008), MHC class I (*P*=0.016), and T-cell coinhibition (*P*=0.020) (Figure 5E). As for tumor immune activation, unlike GMS-high group we noticed that GMS-low group showed higher IFN-γ signaling score (KEYNOTE012, *P*=0.041; POPLAR, *P*=0.021), ICR score (*P*=0.036), inflammation signature score (*P*=0.020), APM score (*P*=0.015), and CD8^+^T-effector score (*P*=0.013) (Figure 5F).

## Discussion

Predictive biomarkers may provide a cost-effective method to identify potential responds to immunotherapy and offer an accurate guide for patients receiving ICI therapy. In this multicohort analysis, we systematically gathered and integrated clinical and genomic data, so that could estimate the connection between genomic signatures and clinical response among patients from NSCLC who had been treated with ICI. We developed and validated a prognostic model based on seven gene mutations in order to predict the survival benefits of patients receiving ICI treatment. According to the present study, GMS divided patients into two different subgroups, with the significant OS and PFS advantage in the GMS-low group. Moreover, the patients with NSCLC in GMS-low group had a better prognosis in the ICI treatment cohort than those in the GMS-high group, and they were independent prognostic factors.

In particular, we utilized the TCGA cohort to characterize tumor immune activation. We found that the GMS-low group can be considered as better immunogenicity, as indicated by higher TMB and an immune-inflammatory phenotype, such as increased CD8^+^ T-cell infiltration. When we used the ssGSEA method to calculate the overall immune cell infiltration level of cancer, GMS-low had significantly higher immune scores than GMS-high, which also confirmed that the GMS-low had stronger immune activity. In addition, the tumor microenvironment (TME) is closely associated with ICI efficacy among NSCLC patients. As the central effector cells in the TME, several studies have suggested that highly infiltrated CD8^+^ T cells were associated with the beneficial prognosis for most tumors, including NSCLC [31]. Peripheral immune cells also play an important role in the antitumor immune response. Increased peripheral CD8^+^ CD28^+^ T cells correlated with favorable survival and better treatment response for patients with NSCLC [32–34]. Studies have indicated that high levels of tumor-infiltrated and peripheral Tregs were related to unfavorable prognosis in NSCLC compared with normal levels [35–37]. Thus, higher TMB, B cells, CD8^+^ T cells, and NK cells, among others, may be the reason that ICI was more effective in patients with GMS-low than in those with GMS-high.

At present, some biomarkers have been found to predict treatment outcomes. MSI analysis could be an appropriate biomarker, while the low incidence of MSI-H in lung cancer might limit its clinical application in this population [38]. Emerging studies indicated that gene mutations including ARID1A, TP53, PBRM1, KEAP1, STK11, NOTCH, and JAK may have different effects for ICI treatment [32–36]. Nevertheless, a single gene mutation may not be adequate to transform the ICI treatment landscape and probably is not a sufficient comprehensive biomarker. For example, TP53 mutations were associated with improved PD-L1 expression and promoted CD8^+^ T-cell infiltration, but the single mutation failed to distinguish sensitive patients with LUAD-receiving immunotherapy. In contrast, the TP53/KRAS comutation combination exhibited a stronger improvement in PD-L1 expression and enhanced tumor immunogenicity compared with the KRAS or TP53 single mutation [39], highlighting the importance of a model that would combine the different genes. We have consequently included most of the possible determinant genes to identify a novel stable GMS to guide patient stratification and personalized treatment.

Currently, PD-L1 and TMB were confirmed to be the primary biomarkers for predicting clinical efficacy of immune checkpoint inhibitors in NSCLC. The ROC curves indicated that the GMS classifier performed better than both TMB and PD-L1. In addition, as PD-L1 and TMB are continuous variables, there is no clear threshold to define whether a response occurs or not. And both biomarkers vary considerably between detection platforms and methods [40,41]. In the present study, seven genes were constructed to form a signature based on mutation data. Nevertheless, risk-scoring formulas and thresholds for mutation-based gene sets can be verified with various methods of tumor analysis, for instance, DNA-sequencing and single nucleotide polymorphism microarray analyses. In this way, mutation-based gene sets are independent from different technical sources, even if various platforms are applied in different centers [27]. Therefore, it is worthwhile to consider a prospective trial to incorporate GMS as a biomarker.

Previous studies have demonstrated the immunological function and prognostic value of seven genes in GMS classifiers for immune therapy. A number of specific genetic variations, for instance, TP53 and STK11, have been proved to create an influence on the infiltration and function of immune cells and the clinical outcomes of ICI therapy [42,43]. As previously reported, the mutation in the DDR pathway can increase the immunogenicity of tumors by accumulating incorrect DNA damage response and promote ICI efficacy [44,45]. There is a possibility that this mechanism contributes to the increased effectiveness of ICIs in patients with ZFHX3 mutations [46]. Moreover, the research indicated that NTRK3 has been proven to be a prognostic biomarker related to TMB and can contribute to the development of bladder cancer immunotherapy [47]. EPHA5 mutation may be used as a biomarker to predict the immune response of patients with LUAD by providing insight into genome-wide mutational burden [48]. Besides, EPHA7 mutations have been demonstrated to be predictive biomarkers for immune checkpoint blockades in several cancer types [49]. MAG has also been proved to be an individual indicator affecting the prognosis of liver cancer, which can be used to guide the effective prognosis and treatment with liver cancer patients [50]. In our study, we integrated genomic mutations associated with ICIs prognosis in NSCLC into a risk model. Therefore, GMS offers a crucial promise in predicting immunotherapy efficacy, and nearly all genes play a key role in regulating the tumor immune microenvironment. The GMS-low can be regarded as an immune inflammatory phenotype, with the activity of immune pathway, higher TMB, and effective immune cell infiltration. Our findings suggested that the GMS may offer critical important insights into the immunological characteristics of NSCLC.

There are several limitations of the present study. First of all, the present study is a retrospective research based on multiple public databases, and the problem of inadequate and limited available data is inherent. The limited size of patients involved in the study may limit application of the conclusions. For example, the sample size for squamous lung cancer was restricted due to the availability of samples in the MSKCC cohort; in the validation cohort, PD-L1 could not be evaluable in some patients due to missing data. Second, our analysis only discussed two of the most important NSCLC subtypes, LUSC, and LUAD, and remaining subtypes were not involved. Finally, our results need to be further verified in future prospective trials and validated through laboratory or clinical trials.

## Conclusion

Ultimately, we explored and validated a novel seven-gene GMS model, which was correlated with better ICI treatment in patients with NSCLC. Therefore, this signature may be used as one of the predictive biomarkers for ICIs. Furthermore, the signature provides a cost-effective approach and a research framework for the construction and evaluation of predictive biomarkers based on immunotherapy in other tumor types. However, the GMS should be validated in future prospective trials and the mechanisms explored in further molecular studies.

## Data Availability

The data that support the findings of the present study are openly available in cBioPortal (http://www.cbioportal.org/), TCGA (https://portal.gdc.cancer.gov/), and UCSC Xena (http://xena.ucsc.edu/).

## Abbreviations

APM: antigen processing machinery
AUC: area under the curve
CR: complete response
CYT: cytolytic activity
DCB: durable clinical benefit
GMS: genomic mutation signature
GZMA: granzyme A
ICI: immune checkpoint inhibitor
ICR: immunologic constant of rejection
iDC: induced dendritic cell
LASSO: least absolute shrinkage and selection operator
LUAD: lung adenocarcinoma
LUSC: lung squamous cell carcinoma
Mb: megabase
MSKCC: Memorial Sloan Kettering Cancer Center
NDB: no durable benefit
NE: not evaluated
NK: natural killer
NSCLC: non-small cell lung cancer
ORR: objective response rate
OS: overall survival
PD-L1: programmed death ligand 1
PFS: progression-free survival
PR: partial response
RCT: randomized controlled trials
SD: stable disease
ssGSEA: single-sample gene set enrichment analysis
TMB: tumor mutation burden
TME: tumor microenvironment
WES: whole exome sequencing
